# Differences in serum and synovial CD4+ T cells and cytokine profiles to stratify patients with inflammatory osteoarthritis and rheumatoid arthritis

**DOI:** 10.1186/s13075-017-1305-1

**Published:** 2017-05-19

**Authors:** Alessandra Penatti, Federica Facciotti, Roberta De Matteis, Paola Larghi, Moira Paroni, Antonella Murgo, Orazio De Lucia, Massimiliano Pagani, Luca Pierannunzii, Marcello Truzzi, Andreea Ioan-Facsinay, Sergio Abrignani, Jens Geginat, Pier Luigi Meroni

**Affiliations:** 1 DISCCO-Department of Clinical Science and Community Health Università degli Studi di Milano, 20122 Milan, Italy; 2 INGM-National Institute of Molecular Genetics “Romeo ed Enrica Invernizzi”, 20122 Milan, Italy; 3 ASST-Gaetano Pini/CTO Orthopedic and Traumatology Specialist Center, Rheumatology and Orthopedic Department, 20122 Milan, Italy; 4 Department of Rheumatology, Leiden University Medical Center, 2300 Leiden, The Netherlands; 5 Laboratory of immuno-rheumatological researches, IRCCS Istituto Auxologico Italiano, 20149 Milan, Italy; 6 Department of Pathophysiology and Transplantation, Università degli Studi di Milano, 20122 Milan, Italy

**Keywords:** Rheumatoid arthritis, Inflammatory osteoarthritis, T helper subsets, Cytokines, Blys

## Abstract

**Background:**

The aim was to investigate CD4^+^T-cell subsets, immune cells and their cytokine profiles in blood and synovial compartments in rheumatoid arthritis (RA) and inflammatory osteoarthritis (OA) to define specific immune signatures.

**Methods:**

Peripheral blood, synovial fluid (SF) and synovial membranes (SM) of RA and OA patients were analyzed. CD4^+^T-cell subset frequencies were determined by flow cytometry, and cytokine concentrations in serum and SF were measured by ELISA.

**Results:**

In peripheral blood, OA patients had altered frequencies of regulatory T-cell subsets, and higher frequencies of Th17 and of Th1/17 cells than RA patients. In the synovial compartment of OA patients, conventional Th17 cells were largely excluded, while Th1/17 cells were enriched and more frequent than in RA patients. Conversely, in the synovial compartment of RA patients, regulatory T cells and Tfh cells were enriched and more frequent then in OA patients. IL-17 and Blys were increased both in serum and SF of RA patients, and correlated with autoantibodies and disease activity. Notably, Blys levels were already significantly elevated in RA patients with low disease activity score in 28 joints (DAS28) and without autoantibody positivity.

**Conclusions:**

Although patients with inflammatory OA have immune activation in the synovial compartment, they display different T-cell subset frequencies and cytokine profiles. Soluble mediators such as Blys might help to discriminate mild clinical forms of RA from inflammatory OA particularly at the onset of the disease.

**Electronic supplementary material:**

The online version of this article (doi:10.1186/s13075-017-1305-1) contains supplementary material, which is available to authorized users.

## Background

Rheumatoid arthritis (RA) and osteoarthritis (OA) represent two manifestations of inflammatory arthritides. Although the etiopathology of these diseases is different, they both involve synovial inflammation [1]. While RA is an autoimmune disease characterized by chronic relapsing-remitting inflammation of the peripheral joints [2], OA is induced by multifactorial mechanisms established primarily by biomechanical stress [3]. Thus, for a long time it has been believed that only patients with RA may have immune cells in the synovial infiltrate. However, recent reports showed that patients with OA also have inflammatory synovial infiltrates that, particularly in the most severe forms, are characterized by high-grade inflammation and could lead to abrupt onset, marked clinical symptoms and structural damage [4]. Those patients with inflammatory OA have inflammatory features that in some instances resemble those of RA. CD4^+^T-cells are key to the initiation and progression of synovitis [2, 5] in patients with RA, and cytokines released into the synovial fluid (SF) by the inflamed tissue likely reflect the composition of effector and regulatory T cells infiltrating the synovia [6]. Relatively little is known about the characteristics of synovial immune cells in patients with inflammatory OA, although recent studies suggest that CD4 + T cells might contribute to the pathophysiology of OA and correlated symptoms, such as pain [7, 8].

Here, we further evaluated the role of CD4^+^ T cells by in-depth analysis of subset composition and the cytokine milieu in peripheral blood and SF during effusion episodes in patients with RA and OA, and of the synovial membrane in patients who also underwent joint biopsy or surgery. We tested correlation between the disease activity parameters and the immunologic alterations in order to determine whether different synovial profiles of immune cells or cytokines could be specifically associated with different types of arthropathy. We report that patients with inflammatory OA display synovial immune activation that presents different signatures in comparison to RA.

## Methods

### Human samples and patients

Buffy-coat blood from 25 healthy age-matched and sex-matched donors (mean age 42 years; age range 26–58 years; male/female ratio 10/15) were obtained from the IRCCS Policlinico Ospedale Maggiore, Milan, Italy. There were 25 patients with RA (mean age 56 years; age range 40–72 years; male/female ratio 12/13) and 18 patients with inflammatory OA (mean age 69 years; age range 57–86 years; male/female ratio 8/10), who were attending the Rheumatology Department of the ASST-Gaetano Pini/CTO Orthopedic and Traumatology Specialist Center of Milan, enrolled in this study. All patients with RA fulfilled the European League Against Rheumatism/American College of Rheumatologists (EULAR/ACR) 2010 classification criteria and their disease activity were assessed using the disease activity score in 28 joints (DAS28) based on the erythrocyte sedimentation rate (ESR) [9]. The median duration of RA was 4.3 ± 3.9 years. We included symptomatic patients with radiographic evidence of mild knee OA (Kellgren and Lawrence grade 2) with an active inflammatory phenotype characterized by the presence of knee effusion synovitis and synovial thickening detected by ultrasound (US) [10]. Clinical, biochemical and cellular data on the enrolled patients with RA and OA are summarized in Table 1.

**Table 1:** Summary of clinical, biochemical and cellular data on patients with RA and OA

Characteristic	Rheumatoid arthritis			Osteoarthritis		
	PB^a^	SF^b^	SM^c^	PB^a^	SF^b^	SM^c^
Number	25	9	4	11	10	9
RF (*n*)^d^	17	9	4			
ACPA (*n*)^e^	17	9	4	-	-	-
DAS28^f^ index, low (0–3) mean 2.42	10/25	0/9	3/4	-	-	-
DAS28^f^ index, medium (3–5) mean 4.26	12/25	6/9	1/4	-	-	-
DAS28^f^ index, high (>5) mean 5.55	3/25	3/9	0/4	-	-	-
ESR^g^ (mm/h)	56.3 (5–87)			10.3 (1–25)		
CRP^h^ (mg/dl)	4.3 (0.4–7.5)			0.27 (0–0.6)		
NSAID^i^ (+/−)	8/25			14/18		
Corticosteroids (+/−)	14/25			0/18		
DMARDs^j^ (+/−)	25/25			0/18		
PBMCs^k^ (10^6^/ml)	1.8 (1.4–2.3)			1.6 (1.3–1.9)		
SFMCs^l^	6.2 (3.6–11.3) *10^5^/ml			9.2 (4.6–18.2) *10^4^/ml		
SMMCs^m^	3.2 *10^6^			1.3 *10^6^		

^a^ *PB* peripheral blood, ^b^ *SF* synovial fluid, ^c^ *SM* synovial membrane, ^d^ *RF* rheumatoid factor, ^e^ *ACPA* anti-citrullinated protein antibody, ^f^ *DAS28* disease activity score based on 28 joint counts, ^g^ *ESR* erythrocyte sedimentation rate, ^h^ *CRP* C-reactive protein (CRP), ^i^ *NSAIDs* non-steroidal anti-inflammatory drugs, ^j^ *DMARDs* disease-modifying anti-rheumatic drugs, ^k^ *PBMCs* peripheral blood mononuclear cells, ^l^ *SFMCs* synovial fluid mononuclear cells, ^m^ *SMMCs* synovial membrane mononuclear cells. *Mean, (values range). ^d^RF nephelometry assay, cutoff value >15 UI/ml. All patients with RA had high positive ^d^RF levels (more than three times the ULN for the laboratory and assay): 264 ± 190. ^e^ACPA: QUANTA Lite® CCP3.1 IgG/IgA ELISA (INOVA Diagnostics), cutoff value >20 UI/ml. All patients with RA had high positive ^e^ACPA levels (more than three times the ULN for the laboratory and assay):125 ± 64. ^h^CRP cutoff value 1 mg/dl.

Peripheral blood (PB) was obtained and collected in heparinized tubes. SF samples from the knee joints of patients with RA and OA were collected during therapeutic needle aspiration into heparinized tubes. SM samples were taken from two patients with RA who also underwent knee joint biopsy and from nine patients with OA and two patients with RA who additionally had knee replacement surgery.

The majority of patients with RA had medium/low disease activity (as summarized in Table 1), and were on medium/low doses of disease modifying anti-rheumatic drugs (DMARDs) (hydroxychloroquine, methotrexate or leflunomide). Thirteen out of twenty-four patients were on low-dose corticosteroid therapy (<8 mg/day). No patient was treated with biological therapy. See Table 1 for details.

### Cell isolation

PB, SF and SM were collected. Peripheral blood mononuclear cells (PBMCs) and synovial fluid mononuclear cells (SFMCs) were directly isolated by Ficoll-Hypaque gradient (Sigma-Aldrich). Synovial membrane mononuclear cells (SMMCs) were isolated by Ficoll-Hypaque gradient (Sigma-Aldrich) after digestion of the tissue with Collagenase Type II (1 ml solution per 40 mg tissue; Sigma-Aldrich) for a variable length of time ranging from 1 h up to 6 h according to the size of the tissue sample.

### Flow cytometry

Single-cell suspension of cells was stained with the following antibodies: CD3 (UCHT1), CD4 (RPA-T4; VIT4), CD8 (RPA-T8; OKT8), CD19 (HIB19), CD56 (B159,), CD127 (eBioRDR5), CD25 (M-A251 BD; BC96), ICOS (ISA-3), CXCR5 (51505), CCR6 (11A9) and CCR5 (27D/CCR5), obtained from BD Bioscience (Becton, Dickinson and Company, NJ, USA), eBioscience (eBioscience Inc, CA, USA), Miltenyi (Miltenyi Biotec GmbH, DE) or R&D (R&D Systems, MN, USA). Samples were passed on a FACSCanto flow cytometer. CD4^+^ T-cell subsets were gated according to well-established phenotypic markers [11]. Thus, Tfh-cells were gated as CD4^+^CXCR5^+^ICOS^+^ [12], Th1 cells as CD4^+^CD127^hi^CD25^−^CXCR3^+^CCR6^−^, Th17 cells as CD4^+^CD127^hi^CD25^−^CXCR3^−^CCR6^+^ [13] and Th1/17-cells as CD4^+^CD127^hi^CD25^−^CXCR3^+^CCR6^+^ [14]. T regulatory cells (Tregs) were gated as CD4^+^CD127^lo^CD25^+^ and Tr1 cells as CD4^+^CD127^−^CD25^−^CCR5^+^CCR6^−^ [15]. Data were analyzed using FlowJo software (Tristar, Palo Alto, CA, USA).

### ELISA

Cytokines in serum and SF were assessed by ELISA. IL-10 was measured with antibody pairs from BD, IL-17-A and IL-21 with antibody pairs from eBioscience (eBioscience Inc, CA, USA). Blys/B cell activating factor (BAFF) was measured using an ELISA kit from Biosupply (BioSupply Ltd, UK).

### Statistics

Statistical significance was calculated using the two-tailed Student *t* test in the case of a Gaussian distribution (determined by the use of SPSS software, IBM); otherwise, the Mann-Whitney test was used for unmatched groups and the Wilcoxon test was used for paired groups. *P* values <0.05 were regarded as statistically significant and are denoted as *p < 0.05, **p < 0.005 and ***p < 0.0005.

## Results

### Patients with inflammatory OA manifest qualitative and quantitative differences in immune cell infiltrates in the synovial compartment compared to patients with RA

**Fig. 1:**
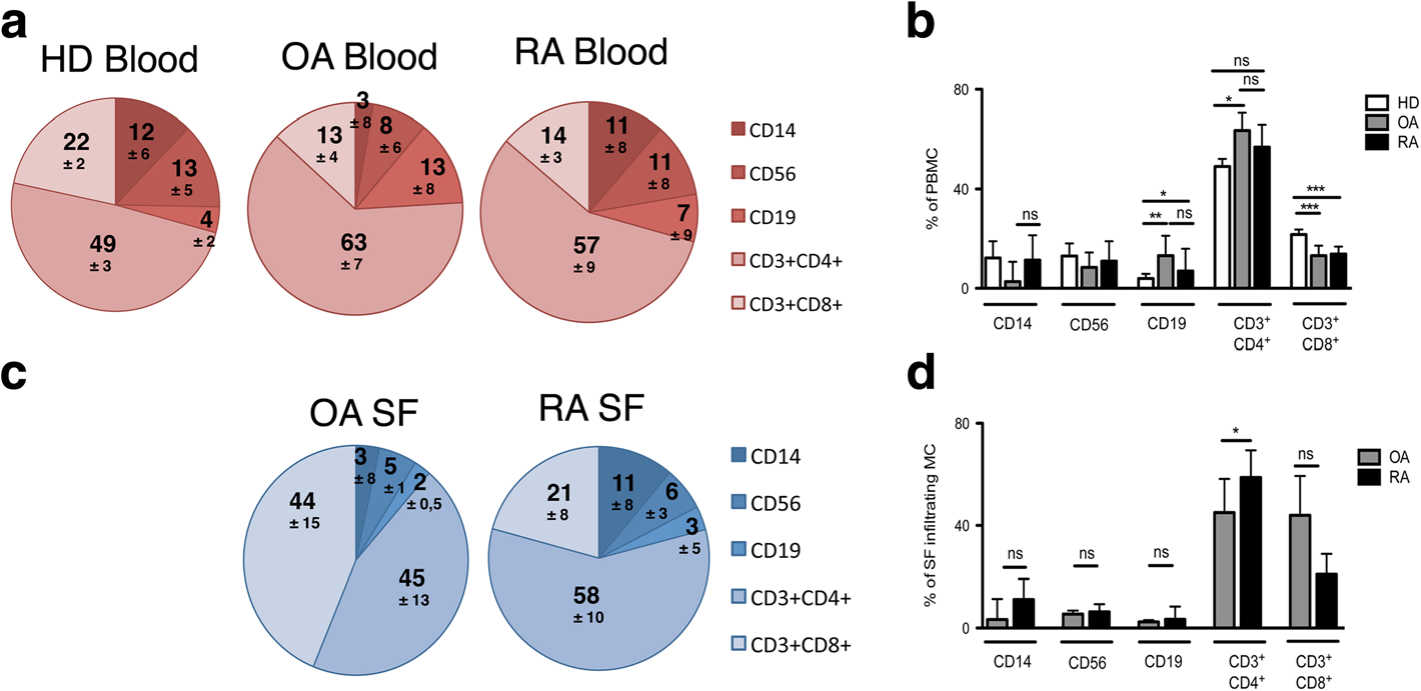
Patients with osteoarthritis (*OA*) manifest qualitative and quantitative differences in the immune cell infiltrates in the synovial compartment as compared to patients with rheumatoid arthritis (*RA*). Frequencies (**a**) and cumulative statistical analysis (**b**) of immune cell populations (monocytes, CD14^+^, B cells CD19^+^, natural killer cells, CD56^+^, CD3^+^ CD4^+^ T cells and CD3^+^ CD8^+^ T cells) in peripheral blood (**a**, **b**) from healthy donors (*HD*, *n* = 25) patients with OA (*n* = 11) and patients with RA (*n* = 24). Frequencies (**c**) and cumulative statistical analysis (**d**) of immune cell populations in synovial fluid (*SF*) from patients with OA (*n* = 6) and patients with RA (*n* = 8): *p ≤ 0.05, **p ≤ 0.005, ***p ≤ 0.0005, Mann-Whitney unpaired two-tailed *t* test. Mean value ± SEM are reported. *ns* not significant, *PBMC* peripheral blood mononuclear cells, *MC* mononuclear cells

To evaluate whether the immune cell compartments in Patients with RA or OA differed from those in healthy donors (HD), we first compared the frequencies and performed statistical analysis of major circulating immune cell subsets, i.e. B cells, CD4^+^ and CD8^+^ T cells, monocytes and natural killer (NK) cells in PB. B cells were more frequent in patients with OA and in those with RA, whereas CD14^+^monocytes were selectively reduced in patients with OA (Fig. 1a and b). Interestingly, although total T-cell frequencies were largely unchanged, patients with RA and those with OA had an increased CD4:CD8 ratio (Fig. 1a, b).

We then analyzed infiltrating immune cells in the SF (Fig. 1c, d) and in the SM (Additional file 1: Figure S1). Immune cells were present in the SF and in the SM of patients with RA and those with OA, as expected [7], and the immune infiltrate was composed mostly of T cells in both cases (Fig. 1c, d and Additional file 1: Figure S1). However, the number of immune cells per milliliter in SF from patients with OA was significantly lower than that in patients with RA (10^4^ vs 10^5^/ml) (Table 1). Moreover, patients with RA had a higher CD4:CD8 ratio in SF (Fig. 1d).

### CD4^+^ T cell subset frequencies in PB and in the synovial compartment distinguish patients with RA from patients with inflammatory OA

**Fig. 2:**
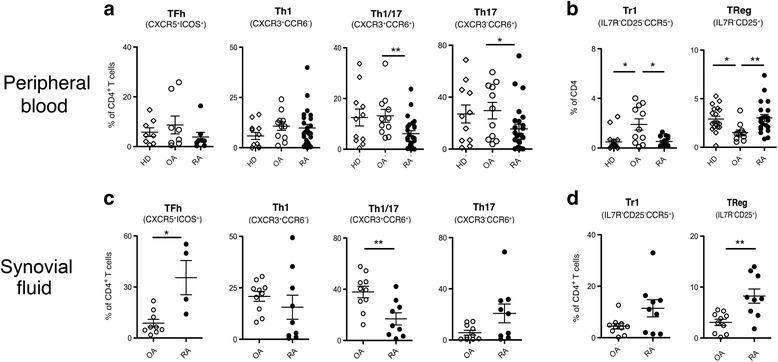
CD4^+^ T cell subset frequencies in blood and synovial fluid distinguish patients with rheumatoid arthritis (*RA*) from patients with osteoarthritis (*OA*). Frequency of CD4^+^ T helper subsets (**a**, **c**) and T regulatory subsets (**b**, **d**) identified according to phenotypic markers in blood and synovial fluid from healthy donors (*HD*) (*open diamonds*), patients with OA (*open circles*) and patients with RA (*closed circles*): *p ≤ 0.05, **p ≤ 0.005, Mann-Whitney unpaired two-tailed *t* test. Mean value ± SEM are reported. *Treg* T regulatory cells, *Th* T helper cells

Next, we compared the frequencies of relevant CD4^+^ T helper and regulatory subsets in PB and in SF from patients with RA and OA, identified according to well-established phenotypic markers [11] (Fig. 2 and Additional file 2: Figure S2).

Patients with RA had lower frequencies of circulating CCR6^+^CXCR3^−^Th17, CCR6^+^CXCR3^+^Th1/17 [12] and of CXCR5^+^ICOS^+^Tfh cells [13] as compared to patients with OA (Fig. 2a), and the reduction in Th1/17 and Th17 cells was statistically significant. Interestingly, patients with OA had significantly increased frequencies of circulating Tr1 cells [15] as compared to both patients with RA and HD, while frequencies of conventional CD25^+^ Tregs were significantly reduced (Fig. 2b).

Consistent with previous studies, we observed an immune infiltrate of T helper cells in the synovial environment of patients with inflammatory OA [16] (Fig. 2c, d). In the SF, patients with OA and patients with RA both had high frequencies of Th1 cells and surprisingly, also of Th1/17 cells, which are thought to play a prominent pathogenic role in autoimmune arthritis [17] (Fig. 2c). Conversely, patients with RA had higher frequencies of Th17 [18, 19] and of Tfh cells than patients with OA (Fig. 2c). However, the frequencies of Th17 cells were low in a fraction of patients with RA, and therefore these differences were not statistically significant (*p* = 0.0625). Patients with RA had also higher frequencies of regulatory T-cell subsets, and the increased frequency of conventional CD25^+^ Treg cells was highly significant (Fig. 2d). Notably, we observed very similar differences in the synovial membranes (SM) collected from the subgroup of patients who also underwent joint needle biopsy or surgery for joint replacement (Additional file 3: Figure S3A/B).

**Fig. 3:**
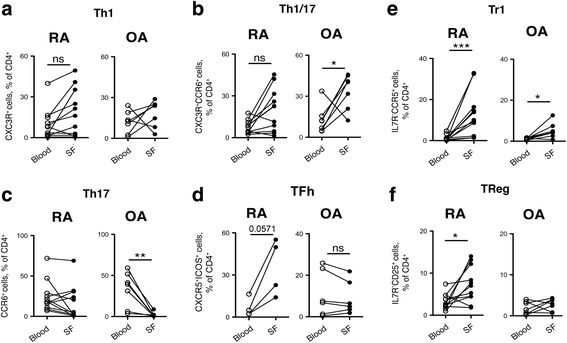
Tissue distribution of T-helper (*Th*) and T-regulatory (*Treg*) subsets in patients with rheumatoid arthritis (*RA*) and patients with osteoarthritis (*OA*). Correlation between Th1 (**a**), Th1/17 (**b**), Th17 (**c**), Tfh (**d**), Tr1 (**e**) and Treg (**f**) subsets in paired samples of blood and synovial fluid (*SF*) isolated from the same patients with OA and the same patients with RA on the same day (values from the same patients are connected by *lines*): *p ≤ 0.05, **p ≤ 0.005, ***p ≤ 0.0005, Wilcoxon paired two-tailed *t* test

We then performed analysis of correlation between T helper and regulatory subset frequencies in paired blood and SF samples (Fig. 3). Interestingly, while no differences were observed in Th1 cells (Fig. 3a), in patients with OA there was significant and strong enrichment of Th1/17 cells in the SF (Fig. 3b), while conventional Th17 cells were largely excluded (Fig. 3c). Conversely, patients with RA did not have significant differences in the frequencies of helper T-cell subsets in PB and SF, with the notable exception of Tfh cells (Fig. 3a-d). Finally, we compared the distribution of T regulatory subsets in blood and SF from patients with RA and OA (Fig. 3e, f). Patients with RA had significantly increased frequencies of both Tr1 (Fig. 3e) and Treg (Fig. 3f) subsets in SF as compared to PB, whereas only Tr1 cells were significantly enriched in SF from patients with OA (Fig. 3d). Notably, there were no significant differences between SF and SM, consistent with the view that T-cell subset distribution in the two synovial compartments is similar (Additional file 3: Figure S3C/D).

### Cytokine levels in serum and SF differ in patients with inflammatory OA and patients with RA, and correlate with disease activity and ACPA positivity

**Fig. 4:**
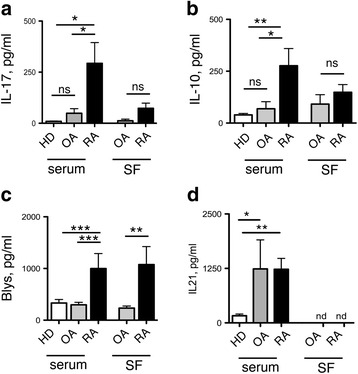
Cytokine levels in serum and synovial fluid discriminate between patients with osteoarthritis (*OA*) and rheumatoid arthritis (*RA*). Blys (**a**), IL-10 (**b**), IL-17A (**c**) and IL-21 (**d**) levels in serum from healthy donors (*HD*) (*n* = 25), patients with OA (*n* = 11) and patients with RA (*n* = 24) and in synovial fluid (*SF*) from patients with OA (*n* = 10) and patients with RA (*n* = 8) patients: *p ≤ 0.05, ** *p* ≤ 0.005, ***p ≤ 0.0005, Mann-Whitney unpaired two-tailed *t* test. Mean value ± SEM are reported. *ns* not significant

Different T-cell subsets preferentially infiltrate the synovium of patients with OA and RA. We asked if their characteristic cytokine products in serum or SF might be useful biomarkers to distinguish patients with RA from patients with OA. We therefore analyzed the prototypical cytokines that are produced by Th17 cells (IL-17, IL-21), Tfh cells (Blys, IL-21) and by regulatory T-cell subsets (IL-10). Notably, however, IL-10 was produced by both IL-7R- and IL-7R^+^ T-cell subsets (Additional file 4: Figure S4), which contain respectively mainly regulatory and helper T cells [11, 15], suggesting that IL-10 is not exclusively derived from regulatory T cells in patients with OA and patients with RA, in particular in the synovial compartment. IL-17A (Fig. 4a), IL-10 (Fig. 4b) and Blys (Fig. 4c) serum levels were significantly elevated in patients with RA but not OA, as compared to HD. Moreover, IL-17A and in particular Blys were also abundant in SF from patients with RA (Fig. 4a and c) [20], but were hardly detectable in patients with OA. In marked contrast, similar amounts of IL-10 were present in SF from patients with RA and patients with OA (Fig. 4b). Interestingly, IL-21 was present in serum from both patients with OA and those with RA, but was completely absent in SF (Fig. 4d).

**Fig. 5:**
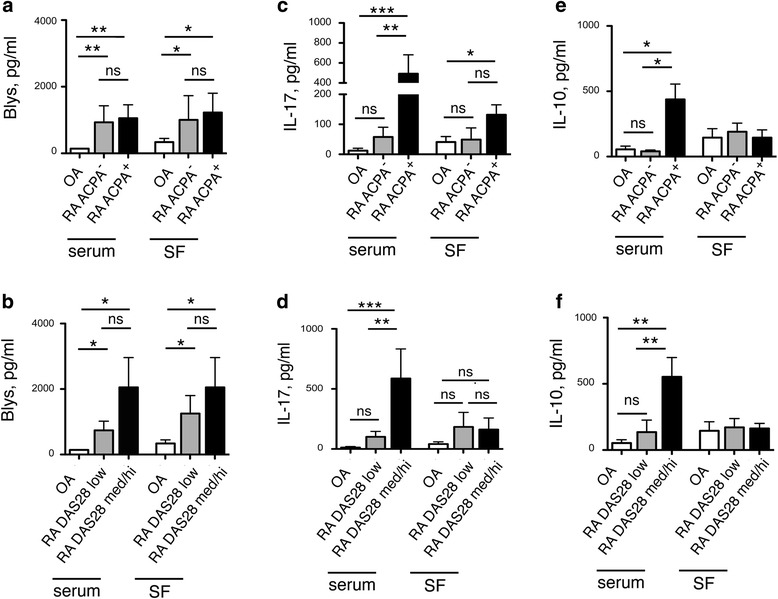
Cytokine levels in serum and synovial fluid (*SF*) correlate with disease activity and anti-citrullinated protein antibody (*ACPA*) positivity. Correlation between serum and synovial fluid levels of Blys (**a**, **b**), IL-17 (**c**, **d**) and IL-10 (**e**, **f**) in patients with rheumatoid arthritis (*RA*) stratified according to ACPA positivity (**a**, **c**, **e**) or to the disease activity score in 28 joints (*DAS28*) (**b**, **d**, **f**), and cytokine levels in serum and synovial fluid from patients with osteoarthritis (*OA*). Cytokine concentrations were determined by ELISA: *p ≤ 0.05, **p ≤ 0.005, ***p ≤ 0.0005, two-tailed *t* test. Mean value ± SEM are reported. *ns* not significant

As cytokine levels in serum and SF differed between patients with RA and OA, we asked if cytokine levels could be useful also to distinguish patients with mild RA from patients with inflammatory OA. Thus, we tested correlation between the levels of cytokines in serum and SF from patients with RA, and the DAS28 and anti-citrullinated protein antibody (ACPA) positivity in these patients and compared them to the levels measured in serum and SF from patients with OA (Fig. 5).

Blys, a cytokine that induces differentiation and proliferation of B cells and promotes the production of autoantibodies [21], was elevated in patients with RA who had a low DAS28, and, surprisingly, independent of ACPA positivity, both in serum and in SF (Fig. 5a, b). In marked contrast, serum IL-10 and IL17A was selectively increased in patients with RA with ACPA (Fig. 5c, e), and/or with an increased DAS28 (Fig. 5 d,f), and there was no significant association between the levels of these cytokines and ACPA positivity or the DAS28 in the SF. A statistically non-significant tendency was observed for increased serum IL-21, ACPA positivity and DAS28 (Additional file 5: Figure S5). Of note, all patients positive for ACPA were also positive for rheumatoid factor (RF) and there were no patients positive for RF only.

As expected, patients with OA had a higher mean age than patients with RA, but on analysis of correlation none of the analyzed parameters were significantly associated with age in the two patient groups (Additional file 6: Figure S6 and Additional file 7: Figure S7). Moreover, we also analyzed a possible impact of corticosteroid therapy, but again found no significant association (Additional file 8: Figure S8).

We concluded that levels of serum cytokine and Blys in the SF could help to distinguish patients with RA from those with OA. Most important, the levels of Blys both in the serum and in the SF might distinguish patients with mild RA from patients with inflammatory OA, whereas elevated serum IL-17A is a more selective feature of severe and/or ACPA-positive patients with RA.

## Discussion

Tissue-specific compartmentalization of immune cells is a distinct pathogenic mechanism that accounts for the selective accumulation of immune mediators in the synovium of both patients with RA and patients with OA. The striking differences in T-cell subset frequencies that we observed in the analyzed patients underline the pathogenic and clinical relevance of the analysis of tissues from the affected joint during active effusion. Indeed, SF samples directly reflect the inflammatory changes in the synovial membrane, and are more easily accessible for research and diagnostic purposes than needle biopsies.

To the best of our knowledge, this study is the first to perform a comprehensive and simultaneous analysis of both helper and regulatory T-cell subsets and their functional products in PB and in the affected tissues of patients with RA or OA. Consistent with previous studies, we observed an immune infiltrate in the synovium of patients with inflammatory OA that contained many Th1 cells [16]. Surprisingly, we also observed strong enrichment of Th1/17 cells, which are thought to play a prominent pathogenic role in autoimmune arthritis [18] and also other organ-specific autoimmune diseases [14]. However, the total cellularity in the SF was lower than in patients with RA, and regulatory T cells were quite frequent.

Consistent with the view that IL-10-producing Treg and/or Tr1 cells control potentially pathogenic IL-17-producing Th1/17 cells in the synovium of patients with OA, IL-10 was quite abundant in SF, whereas IL-17A was hardly detectable. Moreover, CD4^+^IL-7R^−^ T cells, which contain both Tr1 cells and Tregs [11, 15], produced IL-10 in the synovium of patients with OA following polyclonal stimulation. Surprisingly however, we also observed an increase in IL-10-producing IL-7R^+^CD4^+^ T cells, in particular in the synovial compartment and in patients with RA. Interestingly, as compared to patients with OA, there are higher numbers of CCR6^+^IL-7R^+^TH17-cells [18, 19] and CXCR5^+^ICOS^+^Tfh-cells in SF among many but not all patients with RA. Importantly, both subsets can produce IL-10, but provide help to B cells in the production of IgG, and could play a pathogenic role in autoantibody-driven autoimmune disease [22].

Of note, CD25^+^ Tregs and Tr1 cells were highly enriched in SF and in the SM in patients with RA, but were insufficient to control the local production of IL-17A and Blys. We recently demonstrated that both CD25^+^ Treg cells and Tr1 cells can regulate B-cell and T-cell responses, and that Tr1-mediated suppression of T-cell-dependent IgG production is selectively impaired in patients with systemic lupus erythematosus [15]. It seems likely therefore that Tregs and/or Tr1 cells are also functionally impaired in the synovium of patients with RA [23, 24]. In addition, we detected a cytokine storm in serum from patients with RA who had an elevated DAS28, suggesting that the function of regulatory T cells in patients with severe RA is also systemically compromised. Notably, however, while cytokines such as IL-21 and IL-17 are predominantly or exclusively produced by T cells, other cytokines such as IL-10 and Blys are also secreted by myeloid cells or B cells. Indeed, preliminary data suggests that B cells in SF in patients with RA could spontaneously secrete IL-10, indicating that B cells represent a relevant source (data not shown). Blys induces differentiation and proliferation of B cells and promotes the production of autoantibodies [21]. We observed significant increased levels of Blys both in serum and in SF from patients with RA, and virtual absence of Blys in patients with OA. Most important, although Blys serum and SF levels increased with disease activity in patients with RA, Blys was also significantly increased in patients with RA who had a low DAS28, and the levels of Blys were surprisingly independent of the presence of ACPA or RF (data not shown). These findings, generated by the analysis in a cohort of patients with relatively rare inflammatory OA, indicate that Blys might be a useful biomarker to discriminate between mild RA and inflammatory OA at the onset. These findings might help to support the stratification of patients in the future, and provide an additional tool to monitor disease activity at diagnosis and upon subsequent therapeutic interventions.

## Conclusions

Although patients with inflammatory OA have immune activation in the synovial compartment, they display a specific immune signature, which is distinct from that in patients with RA, and this is characterized by different T-cell subset frequencies and cytokine profiles. In addition, soluble mediators such as Blys might serve as biomarkers to discriminate mild clinical forms of RA from inflammatory OA at onset and during acute episodes.

## Additional files

Additional file 1: Figure S1. Frequencies of immune cell populations (monocytes CD14^+^, B cells CD19^+^, NK cells CD56^+^, CD3^+^ CD4^+^ T cells and CD3^+^ CD8^+^ T cells) in the synovial membrane (*SM*) of patients with OA (n = 9) and patients with RA (n = 4): *p ≤ 0.05, unpaired two-tailed Student *t* test. Mean value ± SEM are reported. (TIF 200 kb)

Additional file 2: Figure S2. Gating strategy to identify CD4^+^ T helper and regulatory subsets. **a** Forward and side scatter indicating living lymphocytes; **b** gate to identify CD4^+^ T cells; **c** CD127/CD25 gate to identify memory (CD127^+^CD25^−^), effector (CD127^−^CD25^−^) and Treg cells (CD127^−^CD25^+^); **d** among memory cells (CD127^+^CD25^−^), gates to identify Th1 cells (CXCR3^+^CCR6^−^), Th1/17 cells (CXCR3^+^CCR6^+^) and Th17 cells (CXCR3^−^CCR6^+^); **e** among memory cells (CD127^+^CD25^−^), gate to identify Tfh cells (CXCR5^+^ICOS^+^); **f** Among effector cells (CD127^−^CD25^−^), gate to identify Tr1 cells (CCR5^+^CCR6^−^). (TIF 5405 kb)

Additional file 3: Figure S3. Frequencies and statistical analysis of CD4+ helper (**a**) and regulatory T cell subsets (**b**) among CD4^+^ lymphocytes in the synovial membrane (*SM*) of patients with OA (n = 9) and patients with RA (n = 4): *p ≤ 0.05, unpaired two-tailed Student *t* test. Mean value ± SEM are reported. **c**, **d** Comparison of the frequencies of helper and regulatory T-cell subsets in synovial fluid and membranes in paired samples of the same patients with OA (*lower panels*) or RA (*upper panels*) (values from the same patients are connected by *lines*). (TIF 2769 kb)

Additional file 4: Figure S4. Intracellular IL-10 staining among IL-7R^–^ (**a**) and IL7R^+^ (**b**) CD4^+^ T cells in the peripheral blood of healthy donors (*HD*), in the peripheral blood of patients with OA or RA (*OA Blood*, *RA Blood*), in the synovial fluid of patients with OA or RA (*OA SF*, *RA SF*) and in the synovial membrane of patients with OA or RA (*OA SM*, *RA SM*) following brief polyclonal activation with phorbole ester and calcium ionophore. (TIF 443 kb)

Additional file 5: Figure S5. Correlation of serum levels of IL-21 between patients with RA stratified according to positivity for autoantibodies (*ACPA*) or to the DAS28 disease activity index compared to cytokines serum levels in patients with OA. Cytokine concentrations were determined by ELISA. Unpaired two-tailed Mann-Whitney test. Mean value ± SEM are reported. (TIF 766 kb)

Additional file 6: Figure S6. Correlation between frequencies of CD4^+^ subsets significantly different between patients with RA and patients with OA, and age of patients with RA. Frequency variations in Th17, Th1/17 and TFh CD4^+^ T helper subsets (**a**, **b**) and of Tr1 and CD25^+^ Treg regulatory subsets (**c**, **d**) in peripheral blood (**a**, **c**) and synovial fluid (**b**, **d**) in patients with RA and patients with OA are largely independent of patient age. Pearson’s correlation coefficient: *p* > 0.05 for all. (TIF 439 kb)

Additional file 7: Figure S7. Serum (**a**) and synovial fluid (**b**) levels of IL-10 (*blue graphs*), IL-17 (*light blue graphs*) and Blys (*red graphs*) in patients with RA and patients with OA are largely independent of patient age. Pearson’s correlation coefficient: *p* > 0.05 for all. (TIF 642 kb)

Additional file 8: Figure S8. Distribution of T helper (**a**, **c**) and T regulatory (**b**, **d**) subsets in peripheral blood (*upper panels*) and synovial fluid (*lower panels*) of significantly different subsets in patients with RA according to CS therapy: statistical analysis by Mann-Whitney test, *p* > 0.05. Peripheral blood: (**a**) Th1/17, Th17; (**b**) Tr1 and TReg subsets. Synovial fluid: (**c**) Th1/17; (**d**) TReg subsets. (TIF 607 kb)

## Abbreviations

ACPA: Anti-citrullinated protein antibody
DAS29: disease activity score in 28 joints
DMARDs: Disease-modifying anti-rheumatic drugs
ELISA: Enzyme-linked immunosorbent assay
ESR: erythrocyte sedimentation rate
HD: Healthy donors
IL: interleukin
NK: Natural killer
NSAIDs: non-steroidal anti-inflammatory drugs
OA: Osteoarthritis
PB: Peripheral blood
PBMCs: Peripheral blood mononuclear cells
RA: Rheumatoid arthritis
RF: Rheumatoid factor
SF: Synovial fluid
SFMCs: Synovial fluid mononuclear cells
SM: Synovial membranes
SMMCs: Synovial membrane mononuclear cells
Th: T helper
Tregs: T regulatory cells
US: Ultrasound
