# Supplementary figures and images for: Differences in serum and synovial CD4+ T cells and cytokine profiles to stratify patients with inflammatory osteoarthritis and rheumatoid arthritis
Source: Arthritis Res Ther. 2017 May 19;19:103. doi: 10.1186/s13075-017-1305-1 (PMC5437517; doi:10.1186/s13075-017-1305-1)

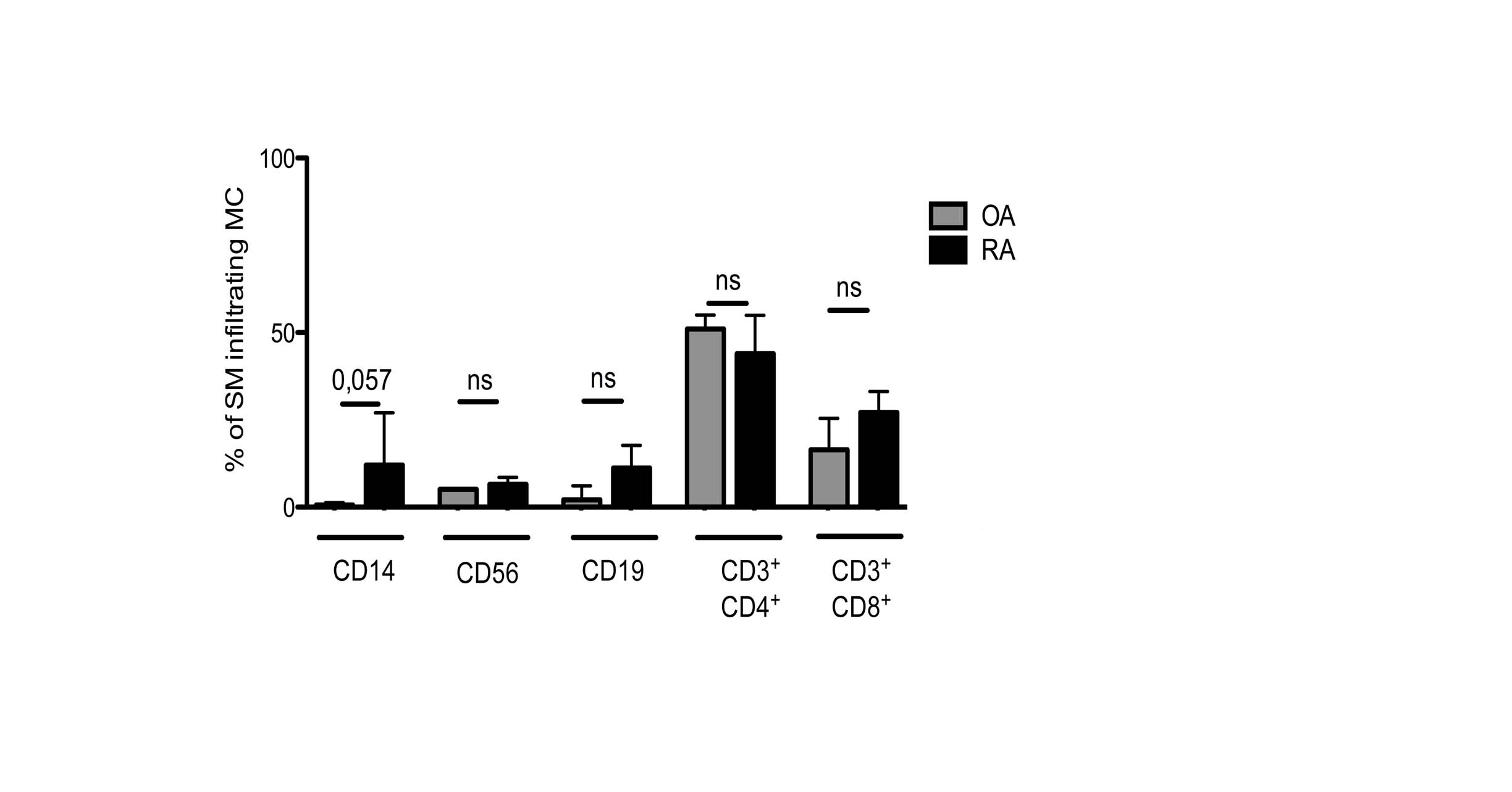

Supplement: Supplementary file 1 — Frequencies of immune cell populations (monocytes CD14+, B cells CD19+, NK cells CD56+, CD3+ CD4+ T cells and CD3+ CD8+ T cells) in the synovial membrane (SM) of patients with OA (n = 9) and patients with RA (n = 4): *p ≤ 0.05, unpaired two-tailed Student t test. Mean value ± SEM are reported. (TIF 200 kb) [file 13075_2017_1305_MOESM1_ESM.tif]

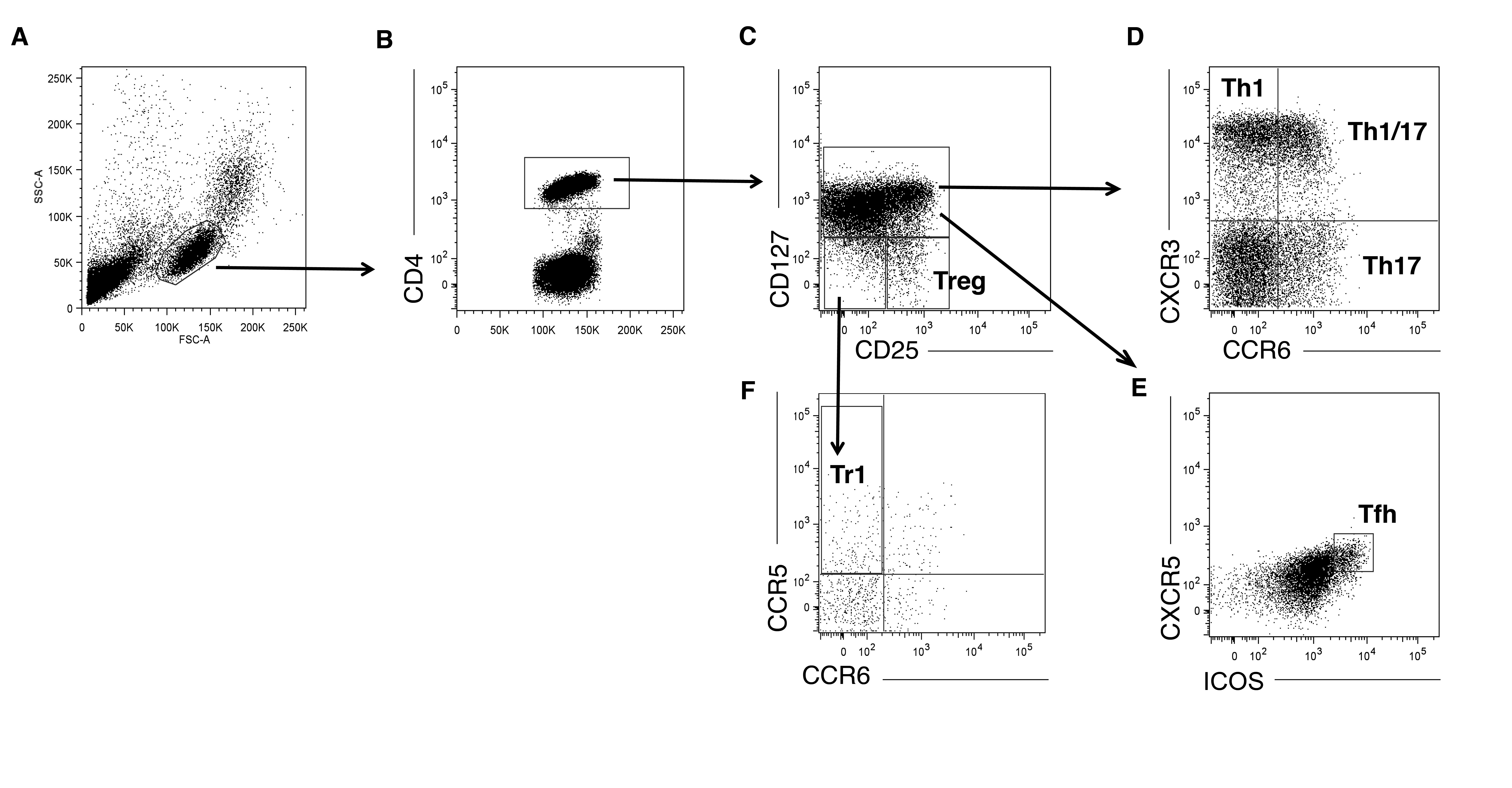

Supplement: Supplementary file 2 — Gating strategy to identify CD4+ T helper and regulatory subsets. a Forward and side scatter indicating living lymphocytes; b gate to identify CD4+ T cells; c CD127/CD25 gate to identify memory (CD127+CD25−), effector (CD127−CD25−) and Treg cells (CD127−CD25+); d among memory cells (CD127+CD25−), gates to identify Th1 cells (CXCR3+CCR6−), Th1/17 cells (CXCR3+CCR6+) and Th17 cells (CXCR3−CCR6+); e among memory cells (CD127+CD25−), gate to identify Tfh cells (CXCR5+ICOS+); f Among effector cells (CD127−CD25−), gate to identify Tr1 cells (CCR5+CCR6−). (TIF 5405 kb) [file 13075_2017_1305_MOESM2_ESM.tif]

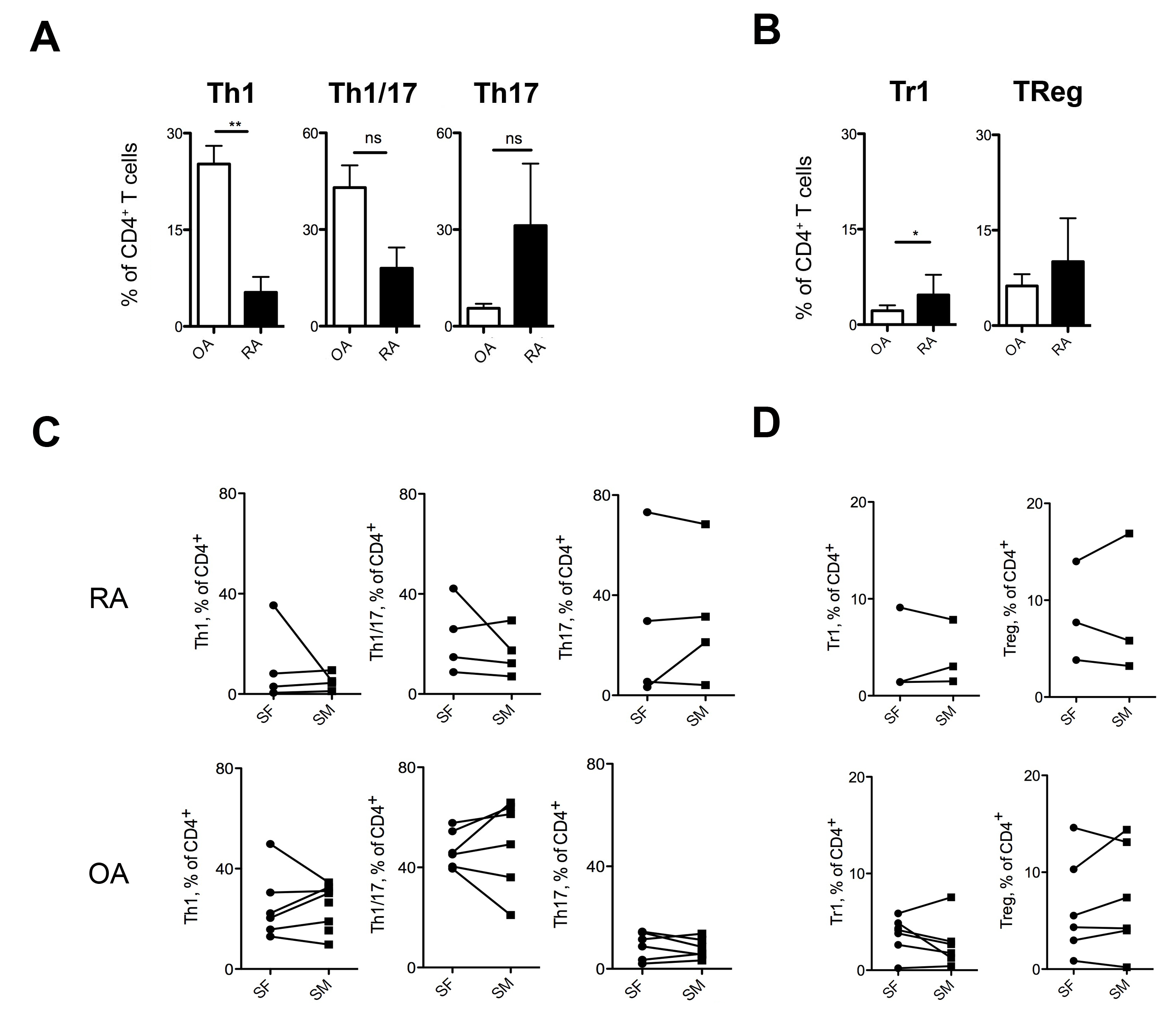

Supplement: Supplementary file 3 — Frequencies and statistical analysis of CD4+ helper (a) and regulatory T cell subsets (b) among CD4+ lymphocytes in the synovial membrane (SM) of patients with OA (n = 9) and patients with RA (n = 4): *p ≤ 0.05, unpaired two-tailed Student t test. Mean value ± SEM are reported. c, d Comparison of the frequencies of helper and regulatory T-cell subsets in synovial fluid and membranes in paired samples of the same patients with OA (lower panels) or RA (upper panels) (values from the same patients are connected by lines). (TIF 2769 kb) [file 13075_2017_1305_MOESM3_ESM.tif]

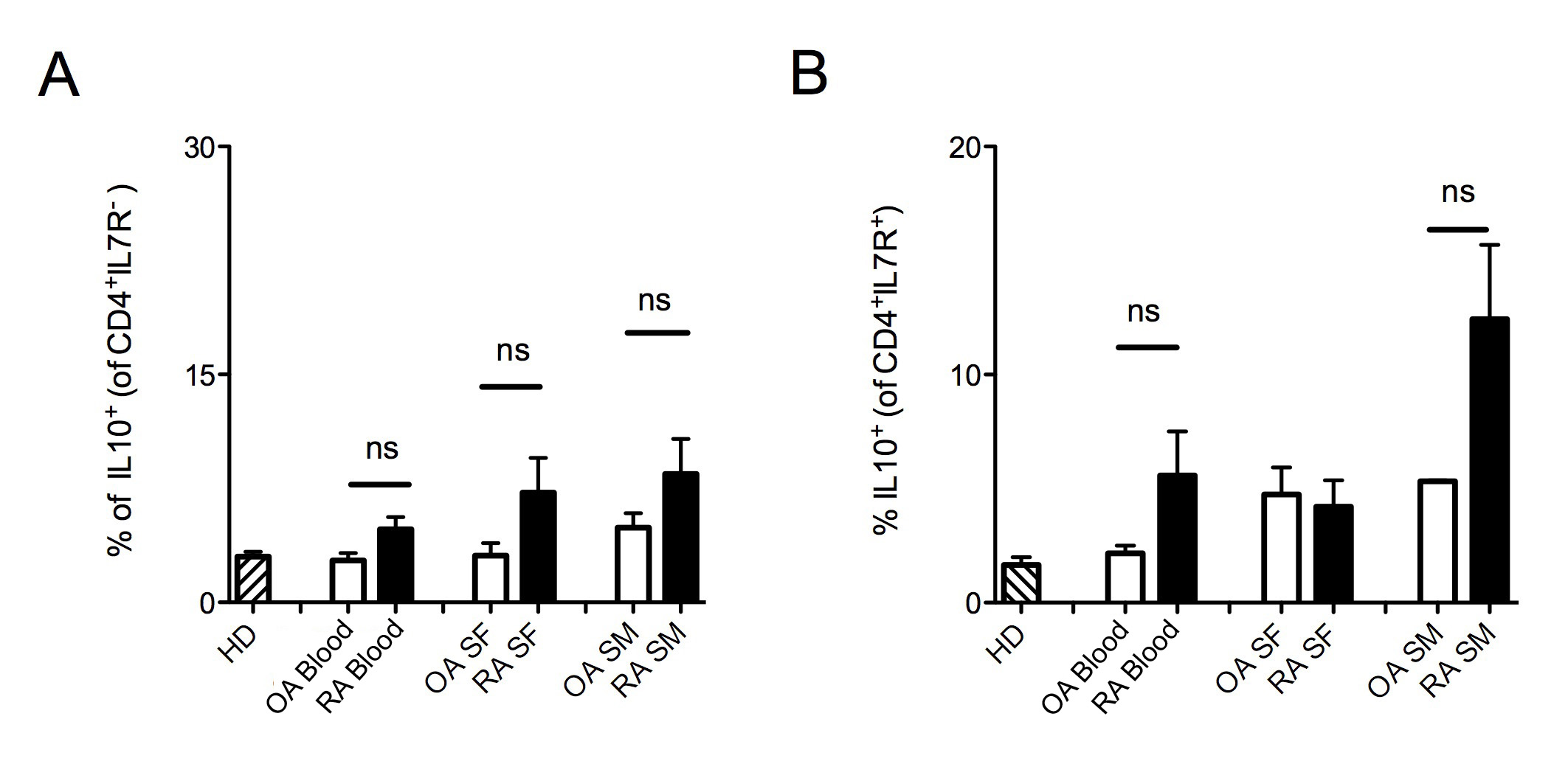

Supplement: Supplementary file 4 — Intracellular IL-10 staining among IL-7R– (a) and IL7R+ (b) CD4+ T cells in the peripheral blood of healthy donors (HD), in the peripheral blood of patients with OA or RA (OA Blood, RA Blood), in the synovial fluid of patients with OA or RA (OA SF, RA SF) and in the synovial membrane of patients with OA or RA (OA SM, RA SM) following brief polyclonal activation with phorbole ester and calcium ionophore. (TIF 443 kb) [file 13075_2017_1305_MOESM4_ESM.tif]

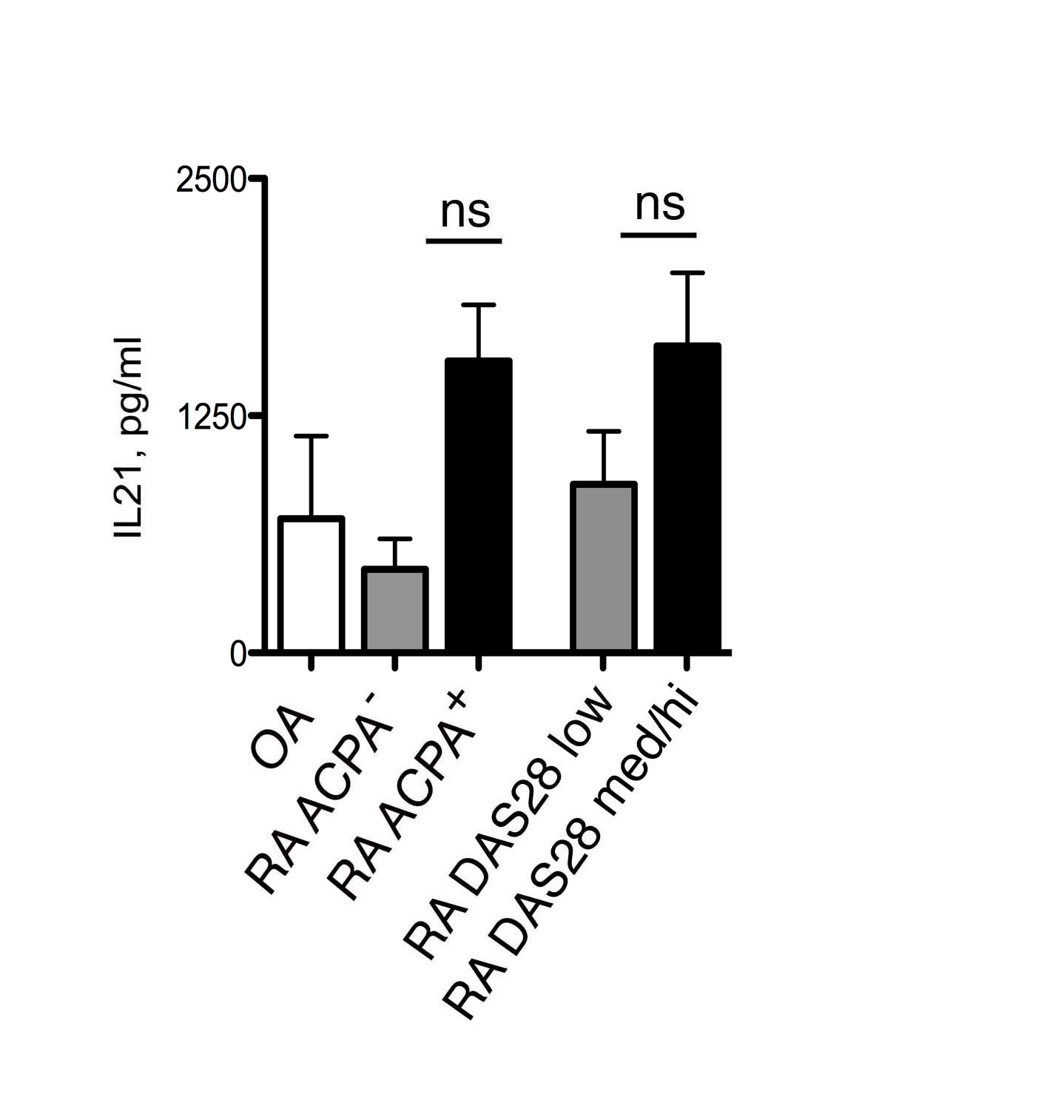

Supplement: Supplementary file 5 — Correlation of serum levels of IL-21 between patients with RA stratified according to positivity for autoantibodies (ACPA) or to the DAS28 disease activity index compared to cytokines serum levels in patients with OA. Cytokine concentrations were determined by ELISA. Unpaired two-tailed Mann-Whitney test. Mean value ± SEM are reported. (TIF 766 kb) [file 13075_2017_1305_MOESM5_ESM.tif]

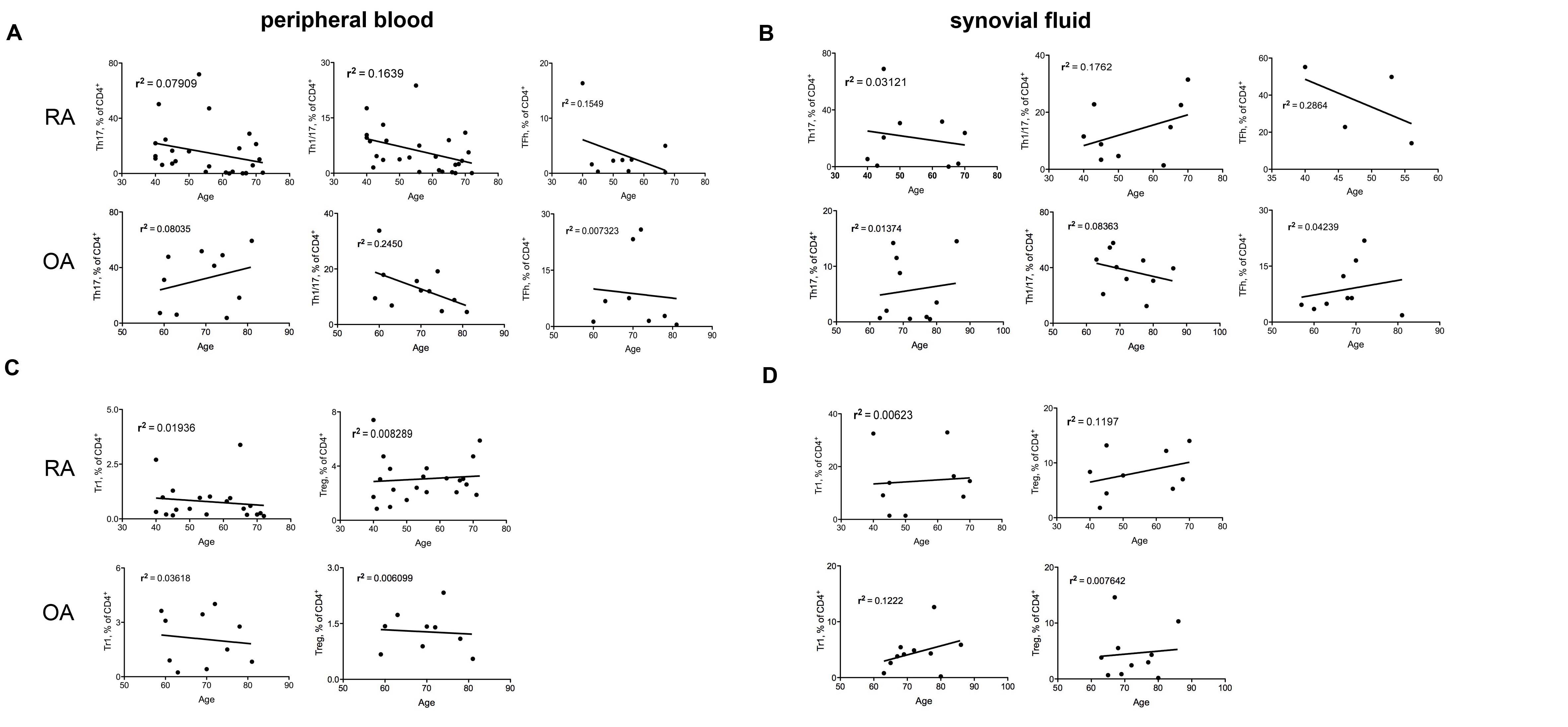

Supplement: Supplementary file 6 — Correlation between frequencies of CD4+ subsets significantly different between patients with RA and patients with OA, and age of patients with RA. Frequency variations in Th17, Th1/17 and TFh CD4+ T helper subsets (a, b) and of Tr1 and CD25+ Treg regulatory subsets (c, d) in peripheral blood (a, c) and synovial fluid (b, d) in patients with RA and patients with OA are largely independent of patient age. Pearson’s correlation coefficient: p > 0.05 for all. (TIF 439 kb) [file 13075_2017_1305_MOESM6_ESM.tif]

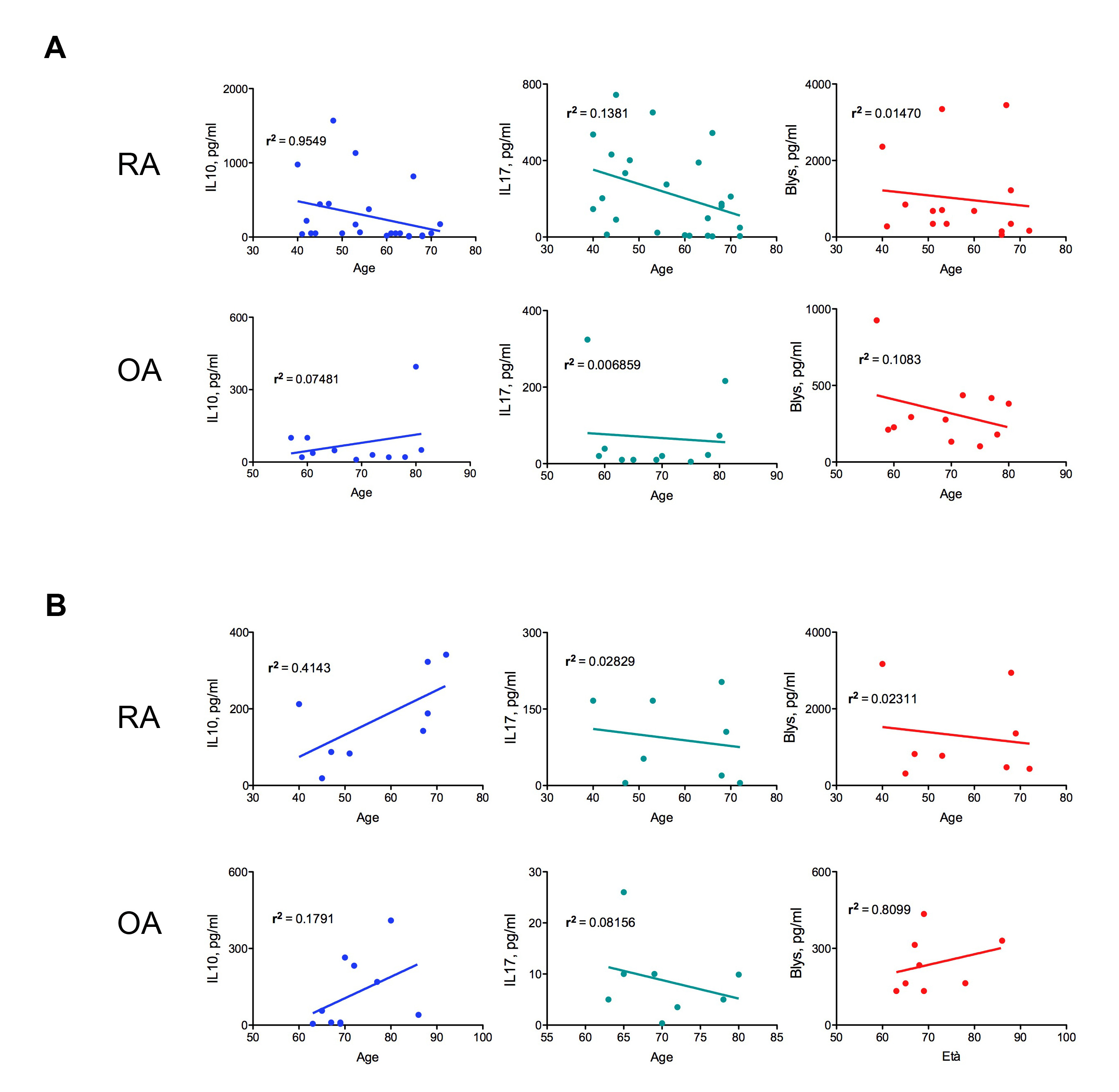

Supplement: Supplementary file 7 — Serum (a) and synovial fluid (b) levels of IL-10 (blue graphs), IL-17 (light blue graphs) and Blys (red graphs) in patients with RA and patients with OA are largely independent of patient age. Pearson’s correlation coefficient: p > 0.05 for all. (TIF 642 kb) [file 13075_2017_1305_MOESM7_ESM.tif]

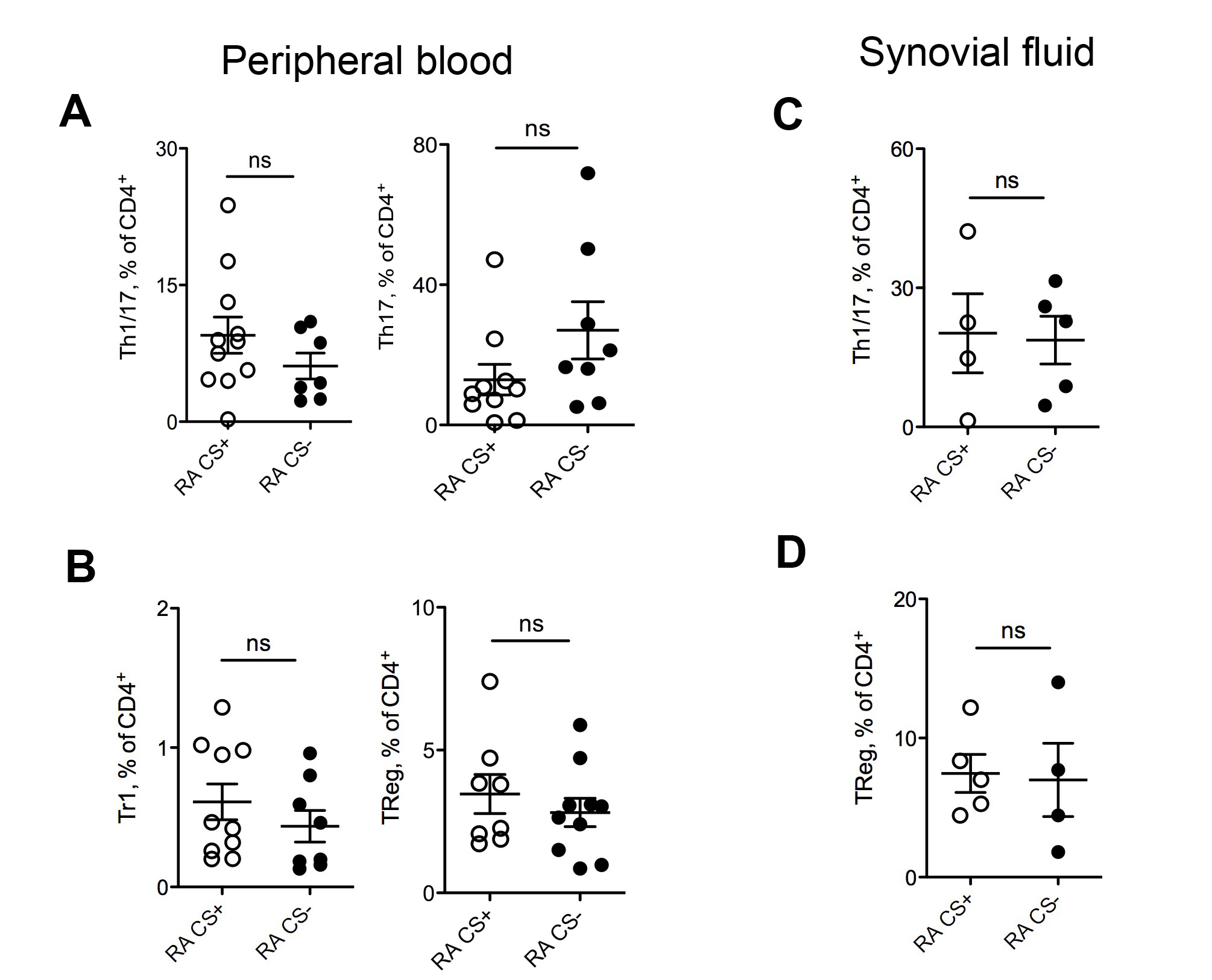

Supplement: Supplementary file 8 — Distribution of T helper (a, c) and T regulatory (b, d) subsets in peripheral blood (upper panels) and synovial fluid (lower panels) of significantly different subsets in patients with RA according to CS therapy: statistical analysis by Mann-Whitney test, p > 0.05. Peripheral blood: (a) Th1/17, Th17; (b) Tr1 and TReg subsets. Synovial fluid: (c) Th1/17; (d) TReg subsets. (TIF 607 kb) [file 13075_2017_1305_MOESM8_ESM.tif]
